# Ethical reflection: The palliative care ethos and patients who refuse information

**DOI:** 10.1177/26323524251355287

**Published:** 2025-07-03

**Authors:** Joar Björk

**Affiliations:** 1 Swedish National Centre for Priorities in Health, Linköping University, Sweden; 2 Department of Research and Development, Region Kronoberg, Växjö, Sweden; 3 Centre for Research Ethics and Bioethics, Uppsala University, Sweden

**Keywords:** palliative care, ethics, communication, truth-telling, autonomy, beneficence, philosophy

## Abstract

Situations wherein a patient refuses potentially important information present tricky ethical challenges for palliative care staff. This critical essay looks to both mainstream bioethics and the palliative care ethos for recommendations on whether or not to provide information in such situations. Such cases highlight controversies surrounding autonomy within mainstream bioethics, making the latter an unlikely source of clear and coherent guidance on this specific topic. The palliative care ethos, as presented by authors within the palliative care community claiming to (re)present such an ethos, may be a more promising source for practical and ethical recommendations. Eleven aspects of the palliative care ethos that may be relevant for such situations are presented, and their implications, individually and collectively, are summarised. Taken as a whole, the palliative care ethos seems to recommend a strategy of using communication skills and time to try to get information across to the patient without forcing things. The recommendation is nuanced and highly contextualised, which increases its validity for clinical practice. Some meta-ethical questions are discussed regarding the use of the palliative care ethos as a source of guidance in ethically challenging clinical situations. All in all, probing the palliative care ethos for practical guidance is an interesting possibility that deserves further ethical and practical reflection.

## Introduction

Palliative care (PC) is a field with myriad ethical challenges.^1^ As many have noted, death and dying (along with pregnancy and birth) offer especially rich soil for ‘big questions’ and ethical reflection. The many relational ethical challenges that arise in meeting and caring for very ill patients further enrich this soil.^2,3^ This brief article will scrutinise one example of the latter kind, presented in the following vignette:

Mr B, a man with disseminated prostate cancer, is being cared for by the specialised palliative care team. He has repeatedly stated to the team that he wishes to have full knowledge about his situation, including what his death may look like. Due to the focus on his many physical symptoms, his questions about death and dying have not yet been answered. Now, the team has set aside time for the conversation Mr B has asked for. But as soon as they start talking, Mr B states that he does not want any more information and that this issue should not be brought up again. He provides no reason. Nothing in the situation appears to have changed. He is perceived to be of sound mind, now as before.

The vignette, drawn from clinical experience but modified to ensure confidentiality, suggests at least two linked ethical challenges: how to respond to Mr B’s expressed preference not to receive ‘any more information’, and how/whether the fact that Mr B previously stated differently should affect the team’s response. The vignette has already been used to empirically study ethical reasoning among PC staff.^4^ The aim of the present article is rather to investigate whether guidance for such a situation can be found within mainstream modern bioethics or the palliative care ethos. The layout of the article is as follows: First, the view of mainstream modern bioethics on such a case is briefly outlined. Second, the concept of ‘the palliative care ethos’ (PC ethos) is explained and defended. Third, normative ideas from the PC ethos that may be relevant to the challenges of the case are highlighted. Fourth, these normative ideas are synthesised to see whether any coherent guidance can be drawn for this particular clinical situation. A reflection on possible implications for PC and mainstream bioethics concludes the article.

## Input from contemporary bioethics

According to the mainstream four-principle paradigm in bioethics,^5^ the challenges in the vignette can be understood as a clash between the dual obligations to respect Mr B’s autonomy and to act in accordance with beneficence. To say anything meaningful about this clash, more *empirical* information is necessary, including what possible benefits, in psychological or medical terms, Mr B might derive from receiving more information. For instance, are there possible future medical interventions that could only be offered after certain information has been exchanged between the team and Mr B? When all relevant empirical data is on the table, some areas of conceptual unclarity remain. Indeed, the vignette brings to light some particularly thorny areas of disagreement within contemporary bioethical thinking. There is disagreement on whether considerations of beneficence should stop at *medical* considerations (however understood) or also include such ‘soft’ values as potential effects on the patient’s psychological coping processes.^6–9^ More complex still is elucidating what guidance could be derived here about respecting patients’ autonomy. Indeed, autonomy is a complex concept that may lead to paradoxical and conflicting recommendations.^10,11^ This is reflected in current competition among different conceptualisations of autonomy for primacy in the bioethical discourse.^12–14^ One contentious issue is how to view situations where people seemingly undermine or curtail their own autonomy, as when Mr B insists on not receiving further information even though this might improve his chances of understanding his situation and making further well-considered decisions. Some conceptions of autonomy suggest that present preferences always trump previous preferences.^15^ Others would investigate the internal relations between Mr B’s different preferences, possibly including higher-level preferences.^16^ Yet others would resist the thought of any easy ordering of ambivalent preferences,^17,18^ leaving it difficult to ascertain which of Mr B’s conflicting preferences to respect in this case. Nonetheless, most mainstream bioethicists would likely emphasise Mr B’s present preferences in this case and conclude that he should not be pushed to communicate, at least in the absence of clear evidence that forcing the conversation with him could provide great medical benefit.^19,20^ To be sure, there are alternatives to the mainstream views in bioethics. Specifically, regarding autonomy, some conceptions stress its relational aspects.^21^ These views, however, require further justification, and even then do not bring greater clarity to the matter at hand. Another contemporary alternative to principlism is virtue ethics.^22^ By stressing professional experience and contextual sensitivity, virtue ethics seems well-aligned with the complex challenges in PC.^23,24^ At the same time, virtue ethics is often criticised for its inability to offer action-guiding recommendations for specific situations, making it less relevant here.^25^ With this in mind, it is time to turn to the PC ethos to investigate whether a recommendation for Mr B’s case can be derived from it, and if so, how it relates to the view in mainstream bioethics.

## Is there a PC ethos and what does it entail?

Texts in and on PC often claim there is such a thing as a special PC philosophy or ethos^26–29^ that is different from – or even stands in opposition to – the ethos of mainstream medicine.^30^ For the purposes of this article, it will be assumed that this claim is factually correct and that a rough understanding of the PC ethos can be provided using texts purporting to present (aspects of) such an ethos written by persons within the PC community. The author has also used his dual background as a specialist physician in PC and clinical ethicist to double-check whether purported aspects can be said to belong to the PC ethos. A further assumption here is that the PC ethos might offer insights relevant to ethical dilemmas in PC, such as that posed by Mr B’s case. It should be underscored, however, that this article neither assumes nor concludes that the PC ethos is itself ethically sound or more ethically relevant than ‘generalist’ ethics guidelines such as the four-principle paradigm mentioned above.^5^ This matter is left for future discussions. For this analysis, only aspects of the PC ethos that might be relevant to the case at hand are included. Thus, the family perspective and cultural sensitivity, although clearly important parts of the PC ethos,^31–33^ are ignored as the vignette makes no mention of next-of-kin or loved ones or of clearly culture-dependent issues. All in all, 11 different aspects of the PC ethos appear relevant to the question at hand. In the following, these aspects will be presented, together with suggestions as to how they might bear on the case. In line with mainstream thinking in bioethics, no single aspect is taken to trump all others; rather, each offers ethically relevant input, which needs to be balanced with the input from the others (see section ‘Attempt at synthesis’).

### Helping towards acceptance and preparedness

One frequently cited aspiration in PC is to help patients prepare for dying and reach a form of acceptance of – if not welcoming – what is happening that might make dying easier.^34,35^ Indeed, what has been called ‘death denial’ is described as a problem that patients should be helped to overcome.^36,37^ For instance, the 6S model for person-centred PC stresses the importance of dying persons’ thinking about the meaning of life and what happens after death.^38^ Although Mr B’s behaviour cannot simply be interpreted as death denial, his reluctance to discuss these matters further may leave the PC team worried about possible denial or at least concerned that this reluctance may hinder them in helping Mr B accept and prepare for his fate. Hence, the normative ideal of acceptance and preparedness provides one reason to continue probing the issue.

### Striving for a good death

Closely linked to the concepts of acceptance and preparedness is the concept of a potentially good, or at least acceptable, death. Indeed, several authors stress that PC practice is predicated on belief in the possibility of a good death and that PC would be quite different if staff held a uniformly dark view of death.^28,36,37,39,40^ Congruent with this is the history of attempts by PC staff and others to change society’s current view of death to a more beneficial outlook.^41–43^ The acceptance and preparedness mentioned above are frequently cited as crucial parts of dying well. In a play on the Latin word *palliare* (‘cloak’), Genuis writes that PC should serve to ‘de-cloak’ the important components of good dying.^28^ The implications for Mr B’s case are once again that staff should not remain passive when it comes to Mr B’s potential grappling with existential issues, even though silence on these matters appears to be Mr B’s current wish.

### Planning ahead

Not only the patient but also the PC team should be well prepared.^37,44^ The notion of ‘hoping for the best, preparing for the worst’^45^ is received wisdom within PC, both as a communicative tool and as a reminder to plan ahead. Many PC instructions underscore the importance of planning for potential minor and major catastrophes further along the disease trajectory.^46,47^ Some plans may be drawn up for Mr B even without his knowledge, but others necessitate his input. The main reason for seeking Mr B’s input relates to autonomy and therefore loses traction if it is accepted that Mr B may have an autonomy-grounded right *not* to be involved in planning ahead. However, there are also practical reasons for seeking Mr B’s input – everyday examples include discussing whether a bed is comfortable enough for future full-time bed care. Hence, the team may feel a need to discuss some things Mr B would prefer left untouched in order to prepare a proper plan.

### Having and using communication skills

Many authors stress that good communication is especially important in PC.^48^ Sometimes the stress is on the importance of PC staff’s having communication *skills*,^49^ but there are also texts that point to the *act of communicating itself* as particularly important in PC. For instance, Reich’s concept of ‘expressive suffering’ and the metaphor of ‘bearing witness’ are frequently quoted in PC literature, with the implication that it is desirable for a particular form of communicative interaction to take place between patient and PC staff.^50,51^ Conversely, writers speak derisively of a ‘conspiracy of silence’, referring to situations where staff know the patient is dying but do not tell him/her, making preparation for death impossible.^35^ The implication for the present context would be, therefore, to view Mr B’s withdrawal from (some forms of) communication with caution and not simply abide by his plea to leave certain things undiscussed. At the same time, having communication skills may make it easier to pursue even unwanted conversations. Indeed, the insistence that PC staff need extraordinary communication skills might even be taken to contain an indirect argument that PC staff should *not* remain silent in situations such as this, as doing so would not require any particular skills.

### Understanding patients’ true values

PC prides itself on being especially apt at elucidating, and being attuned to, what patients *really want*.^52^ This reflects the view that PC staff should strive to gain a proper understanding of patients and their values. Relatedly, the PC ethos is keenly aware that patients are sometimes misunderstood within healthcare. Such misunderstanding may stem from staff’s having preconceived views of patients or stopping at a form of surface understanding.^48,53^ Indeed, it has been suggested that the phrase ‘What is important to you?’ is especially important in PC and is a good conversation tool to help patients focus on what really matters, as this may sometimes not be immediately clear, even to the patients themselves.^54–56^ In terms of Mr B’s situation, this would suggest that it is important for the team to understand why Mr B has changed his mind and now desires no further information.

### Holistic care

Taking a holistic approach, or providing holistic care, is a core tenet of PC.^34,57,58^ It is not immediately evident what approach would be the most holistic in Mr B’s case, but it could be argued that it is more holistic to take all his (known) preferences into account than merely to let his current preferences eclipse his previous ones. This argument would support a more proactive stance when it comes to talking to Mr B, even about those things he presently refuses to discuss (as he previously expressed a preference for thorough communication). However, another way of operationalising the ideal of holism might be to consider all the other possible subjects to discuss with Mr B besides the one he refuses to discuss. Indeed, empirical evidence suggests that many patients who do not wish to partake in treatment-related decisions may still wish to be involved in other forms of shared decision-making.^59^

### Building relationships

PC places special emphasis on building relationships and trust with patients.^44,60^ Indeed, empirical studies suggest that PC staff consider relationship-building their most important priority.^61^ In this case, prioritising the relationship may engender an argument not to push things with Mr B as this might damage the relationship. At the same time, it could be expected that relationship-building, over time, might later enable PC staff to talk about things that today cannot be discussed. The upshot here, then, is that staff may later succeed in providing information, even if they fail to do so today.

### Promoting dignity

Although in bioethics, the notion of dignity is frequently criticised for being too vague to be put to any normative use,^62^ dignity figures prominently in the PC ethos (Some authors, however, have criticised the usage of ‘dignity’ in PC).^37,46,63^ Indeed, many writers articulating the PC ethos, such as the authors of the previously mentioned 6S Model, stress the profound vulnerability of PC patients and the importance of the resulting threats to their dignity.^38^ Implications of the value of dignity in PC include being respectful towards patients and loved ones and helping patients maintain a sense of self, control, and value despite physical decline.^64,65^ Here, it seems natural to suggest that caring about dignity speaks against expressly overriding Mr B’s stated will and for allowing Mr B to feel that he is in control as much as possible.

### Everything changes

The crude reality of PC is that most patients are on a downward trajectory and undergoing rapid changes. Many of these changes are physiological, but the PC ethos also emphasises that ambivalence and existential turmoil are common for patients and families.^66–68^ The relevance for the present case is that PC staff should not be surprised that Mr B has changed his mind, nor should they be if he changes his mind yet again. As with section ‘Promoting dignity’, this suggests that PC staff may allow themselves to postpone difficult conversations as there may come times when Mr B is more ready.

### Focus not on medical interventions

While the PC ethos certainly prioritises symptom control,^27^ it gives less priority to other forms of medical intervention than those aiming at symptom control.^69,70^ Indeed, empirical studies suggest that staff perceive focusing on medical interventions as sometimes crowding out other, arguably more important, issues in a patient’s last phase of life.^71,72^ The catchphrase ‘*being with* rather than *doing for*’ captures this aspect of the PC ethos, which deliberately downplays perceived low-value interventions and naive medical heroism.^73^ In the current context, stressing issues other than medical intervention may ease the worry that Mr B’s present unwillingness to discuss the future makes some potential interventions impossible (see section ‘Input from contemporary bioethics’).

### Adapting and improvising

Empirical studies indicate that PC staff regard adaptability and the capacity to improvise according to the individual patient’s situation as core PC virtues.^74–76^ Indeed, one can hypothesise that the need to navigate and balance the many values evident in this article (plus some others) is in itself a driver of flexibility for PC staff. As for the situation with Mr B, stressing adaptability may mean that staff should work around the problem rather than confronting it head-on, perhaps by finding new ways to interact with Mr B or by helping him in ways that do not require the delivery of unwanted information.

## Attempt at synthesis

All in all, 11 relevant aspects of the PC ethos (‘Helping towards acceptance and preparedness’, ‘Striving for a good death’, ‘Planning ahead’, ‘Having and using communication skills’, ‘Understanding patients’ true values’, ‘Holistic care’, ‘Building relationships’, ‘Promoting dignity’, ‘Everything changes’, ‘Focus not on medical interventions’ and ‘Adapting and improvising’) have been presented. As noted, these provide seemingly contradictory recommendations for Mr B’s case. The first five suggest that Mr B’s express wish not to discuss his future disease trajectory should be contravened, or at least not simply obeyed. ‘Holistic care’ provides arguments in both directions, and the next three suggest that Mr B’s express wish should be honoured. ‘Focus not on medical interventions’ serves to somewhat attenuate one possible problem with honouring Mr B’s wish, while ‘Adapting and improvising’ provides general advice for interacting with patients, which might be useful whatever strategy one chooses. So, how is anybody to make sense of this?

True to the attention to detail and context characteristic of PC^38,77^ and to taking seriously the complexities of the human condition, suffering, and ethics, one should resist the temptation to reduce the ethical challenge in Mr B’s case to an easy dichotomy like ‘should Mr B be informed whether he wants to or not?’ Reductionistic tendencies and overly abstract reasoning hamper the clinical relevance of bioethics.^78^ Anybody working in patient care knows there are a thousand options between being silent and gushing out all available information as if emptying a cup. With the admonition to steer away from easy reductions in mind, it may indeed be possible to construct a coherent clinical recommendation from the puzzle pieces offered by the PC ethos. Starting at the ‘emptying a cup’ end, there is a lot within the PC ethos (‘Promoting dignity’, ‘Building relationships’, ‘Having and using communication skills’) that strongly warns against running roughshod over Mr B’s current expressed preferences. Hence, advocating a clear and direct confrontation would *not* be in line with the PC ethos. However, there is also plenty of support in the PC ethos for *at least trying to* get through to Mr B with some of the information, at least at some point (‘Helping towards acceptance and preparedness’, ‘Striving for a good death’ and ‘Planning ahead’). Of the latter, ‘Planning ahead’ is quite uncontroversial, whereas the potential paternalism of ‘Helping towards acceptance and preparedness’ and ‘Striving for a good death’ has been a topic for debate even within the PC community,^46^^(chap 2.2)^ suggesting that these goals are better seen as aspirational than as absolute. As a further implication of ‘Planning ahead’, one may suspect that the discussion Mr B presently wishes to avoid is related precisely to existential issues like preparedness and dying well, rather than to practical issues. Hence, there may be an opening for the PC team to use their communication skills to get to the practical matters without going for the existential bulls-eye. The PC wisdom captured in ‘Everything changes’ encourages a form of attentive, yet laid-back, expectancy, and may in itself be encouraging to PC staff – indeed, everything need not be done at once, and the rules for communication need not be set in stone.

## Reflection

Scrutinising Mr B’s case from the dual perspectives of mainstream bioethics and the PC ethos suggests some subtle differences (i.e., if the assumptions and arguments put forth in the article are accepted. In the spirit of dialogue, readers are heartily invited to criticise this article. Perhaps there *is* no PC ethos? Has the author misunderstood any aspect? Are important aspects which might have turned the synthesis in another direction missing?). Both sources agree that the patient should not be forced into any form of discussion *at the present moment*; however, whereas mainstream bioethics would likely let things rest there – unless Mr B brings the matter up again – the PC ethos suggests a much more forward-leaning professional approach. Also, the PC ethos takes into account an intentionally wide range of considerations, some of which do not neatly fit into, for instance, the dominant principlism espoused by Beauchamp and Childress and may therefore be lost in mainstream analysis. Examples include ‘Helping towards acceptance’, which could be included in ‘beneficence’ but is seldom contemplated in mainstream bioethics, and ‘Having and using communication skills’, which is generally taken for granted (and therefore, again, lost from sight) in mainstream bioethics.

If there are indeed subtle differences between the recommendations generated from mainstream bioethics and those generated from the PC ethos, this raises important practical and meta-ethical questions. First, where does the PC ethos stand in relation to mainstream bioethics? Beauchamp and Childress themselves advocate the ‘specification’ of general bioethical terms^5^; could the PC ethos then perhaps be seen as an attempted *specification* of general bioethics to suit the PC context? If so, this could be used to counter the common criticism that general principles are too vague to be put to clinical use.^78,79^ Another possible approach would be to argue, as some have,^80,81^ that PC is *different* from other spheres of morality and therefore merits its own ethics. This, of course, would necessitate a sound theory of different vs overlapping spheres of morality in healthcare. Regardless of whether the PC ethos deviates from mainstream bioethics because it is a specification of it or because it is an alternative to it, the ethos itself needs to be subject to the same scrutiny as, for instance, the four-principle paradigm. Indeed, PC staff cannot accept the PC ethos just because it ‘feels right’ (or even worse, because it ‘feels familiar’). Indeed, for a practice leaning as heavily on values as PC does, continuous self-criticism addressing the underpinning values is of utmost ethical importance.^82^ Two possible points of departure for an ethical criticism of the PC ethos, suggested by the present contemplation, are as follows: (1) PC prioritises ‘Understanding patients’ true values’. So far, so good – any ethical theory would agree. But empirical studies indicate that PC staff may sometimes become overzealous in their attempts to discern patients’ values.^4,83,84^ Where is the line between being attentive to patients’ values and snooping or demanding that the patient open up like a book? (2) If it is true that PC is predicated on an ideal of the good – or at least good enough – death, how can PC staff avoid turning their principled aversion to *attitudes* like ‘death denial’ against *individual patients* such as Mr B who may be taken to express death denial?

The analysis prompts a further, somewhat mundane question. Mr B’s case hinges on, among other things, the possible values of ‘Acceptance and preparedness’ and ‘Striving for a good death’. This makes the lack of good empirical evidence regarding these values acutely concerning. Imagine, by way of analogy, the alarm it would stir if surgeons did not know the odds of success for their preferred surgical interventions. It is promising that empirical data is beginning to accumulate,^85,86^ but we are still a long way from being able to say anything about the value *to some specific person* of coming to ‘acceptance’ (whatever this even means). Even more relevant to Mr B’s case, what we know about the value of acceptance *to those who have accepted* cannot easily be translated into an estimate of the (possible) value of *forcing* acceptance on somebody (if such a thing is even possible). In short, PC’s empirical blind spots are an ethical concern, and there is good ethical reason to disagree with those who claim that measurement is inappropriate for PC.^70^

## Conclusion

Situations in which a patient refuses potentially important information present tricky ethical challenges for staff in PC. This article has investigated whether recommendations for such situations can be found in mainstream bioethics and/or the PC ethos. The first conclusion is that none of the sources provide straightforward and consistent recommendations on this topic. Second, to the extent that recommendations can be inferred, the two sources seem to provide partly contradictory recommendations. Third, by putting 11 aspects of the PC ethos together, one may derive a comprehensive and nuanced recommendation that is contextually sensitive. This suggests that the PC ethos may be a rich and relevant source of ethical guidance in tricky clinical situations. At the same time, the PC ethos must not be followed uncritically, and potential clashes between mainstream bioethics and the PC ethos present an obvious starting point for further enquiry. Some critical comments regarding the PC ethos and its application have been provided. All in all, this ethical reflection presents compelling indications that it may be worthwhile to double-check the PC ethos against both real-life situations and insights from mainstream bioethics.
